# Editorial: Antimicrobials in Wildlife and the Environment

**DOI:** 10.3389/fmicb.2021.783118

**Published:** 2021-11-05

**Authors:** Ana de la Torre, Alain Hartmann, Astrid Louise Wester, Rustam Aminov

**Affiliations:** ^1^Centre for Animal Health Research (CISA), National Institute of Agricultural and Food Research and Technology (INIA), Spanish National Research Council (CSIC), Madrid, Spain; ^2^Agroécologie, AgroSup Dijon, INRAE, Université de Bourgogne, Université de Bourgogne Franche-Comté, Dijon, France; ^3^Department of Bacteriology, Norwegian Institute of Public Health (NIPH), Oslo, Norway; ^4^The School of Medicine, Medical Sciences and Nutrition, University of Aberdeen, Aberdeen, United Kingdom

**Keywords:** antimicrobial resistance, antimicrobial residues, environment, wildlife, food chain

Antimicrobial resistance (AMR) is a major threat to both human and animal health since it significantly diminishes the therapeutic options available. Significant amounts of the antimicrobials used in humans and animals are excreted into the environment essentially unchanged, or as metabolites that still retain antimicrobial activity. They reach the environment via direct excretion by pasture animals, from discharges from wastewater treatment plants, via application of manure and sludge to agricultural fields, or direct application of antimicrobials in aquaculture. The release of antimicrobials into the environment can exert a selective pressure on environmental microbiota, leading to the selection of antimicrobial resistance genes (ARGs) in a variety of ecosystems. Thus, the microbiota of plants, wild animals and the environmental microbiota in general can be affected. Another way of dissemination of ARGs into the environmental ecosystems is via AMR bacteria in animal and human faeces. The resulting pool of ARGs in the environment can be considered as self-replicating genetic pollutants, with the possibilities of horizontal transfer, recombination, and generation of a broader diversity of ARGs. From this pool, ARGs can be reintroduced back to human and animal pathogens, thus contributing to the global problem of AMR.

Antimicrobial residues and AMR determinants have been reported in soils, surface and groundwater, glaciers, air, agricultural produce, wildlife and other urban, agricultural and natural environments. The main aim of this Research Topic was to uncover the role of AMR in wildlife and the environment for better understanding of AMR epidemiology in a globalised world. Within this topic, 11 articles have been published that complemented our knowledge on the occurrence and diversity of antimicrobial residues, AMR bacteria and ARGs in the environment and wildlife.

Mbanga et al. characterised genomic diversity of environmental *E. coli* isolates from a wastewater treatment plant and surrounding receiving waters in South Africa using phylogenomic analysis. They concluded that these isolates mainly clustered with clinical isolates, thus highlighting their importance for public health. Continuous surveillance of AMR bacteria in wastewater and associated surface waters could serve as a proxy for local AMR and its dynamic over time. Such a surveillance strategy may be highly relevant in low-resource settings, where clinical surveillance of AMR is too costly. The dissemination of AMR bacteria from river water to vegetables irrigated by this water was studied by Díaz-Gavidia et al. in Central Chile. They isolated Enterobacterales strains that were resistant to four antibiotic classes, with some of them demonstrating multi-drug resistant (MDR) phenotypes. The occurrence of MDR Enterobacterales during the rainy season was less frequent compared to the dry season. Association of AMR frequency with season and vegetable type contributes to a better understanding of a potential public health impact of AMR/MDR bacteria present in irrigation water and vegetables.

Jauregi et al. evaluated the potential risks associated with antibiotic residues, ARGs and mobile genetic elements (MGEs) introduced into soil and crops via cow manure application. Authors compared different treatments (slurry, fresh, or aged manure from conventional and organic livestock farms) and crops (wheat and lettuce) but no single treatment could be identified as superior to the others to reduce the potential resistome risks. The authors concluded that the treatments should be specific and take into consideration the amendment, soil and crop types.

AMR can be also exchanged between humans and animals through pests and wild animals. The role of common pests in poultry such as the lesser mealworms to serve as a potential reservoir of zoonotic *Salmonella enterica* strains was investigated by Donoso et al. They isolated 15 *S. enterica* strains, 14 of which belonged to the Infantis serotype, with the carriage of pESI plasmid and MDR phenotype.

Wildlife may also contribute to the dissemination of genes conferring resistance to clinically relevant antimicrobials. They could serve as reservoirs of AMR bacteria and also represent epidemiological links between human, livestock and natural environments, especially in the case of long-distance haulers such as migratory birds. Several articles in the Research Topic addressed this issue. Haenni et al. investigated 424 wild birds and 16 wild mammals in a rescue centre in France for the presence of AMR bacteria. They demonstrated a wide dissemination of Enterobacteriaceae with an IncHI2/ST1 plasmid carrying the blaCTX–M−9, blaSHV−12 and mcr-9 genes. Interestingly, these clones are not *bona fide* microbiota of birds but, similarly to nosocomial infections in hospitals, represent dissemination of clones within the rescue centre. These findings suggest a potential dissemination of ESBL-positive clones to the natural environments from rescue centres. Plaza-Rodríguez et al. investigated the occurrence and AMR patterns of several bacterial species in certain wild animals in Germany, including wild boars, roe deer, and wild ducks and geese. In general, the prevalence of AMR bacteria was low. However, resistance was identified against clinically relevant drugs such as third-generation cephalosporins, fluoroquinolones and colistin. Skarzyńska et al. characterised the AMR status of 71 *E. coli* isolates from free-living birds in Poland using phenotypic assessment and WGS. Multiple resistance types were found, including those towards cephalosporins, quinolones, polymyxins, aminoglycosides, as well as fosfomycin. Molecular epidemiological analyses revealed that the *E. coli* strains were of the global lineages ST131, ST10, and ST224 as well as the three novel STs, 11104, 11105, and 11194. O'Hagan et al. investigated the AMR prevalence in *E. coli, Salmonella* spp., and methicillin resistant *Staphylococcus aureus* (MRSA) isolates from European badgers and red foxes in Northern Ireland. No MRSA were detected, while ESBLs were detected in 8.90% of badger and 11.53% of fox faecal *E. coli* isolates, which, in addition, also displayed MDR phenotype. AmpC type resistance was found only in the *S. enterica* subsp. *arizonae* isolate. Detection and quantification of ARGs in the gut of kelp gulls and Magellanic penguins revealed that AMR profiles differ between these two bird species (Ewbank et al.), which could be reflective of differential biology, ecology and proximity to human-impacted areas. In another study, the distribution and resistance to third-generation cephalosporins in *E. cloacae* complex members and their relationship between wild anoles and human activities (Pot et al.) was investigated. No relation was identified. The authors suggested that the high level of resistance in wild anoles was probably due to environmental factors that favour the selection of these resistant strains.

Other antibiosis factors like polyene produced by *Streptomyces* that occur naturally in the environment can be active again pathogenic fungi, and constitute an alternative to chemical fungicide treatment and might participate to reduce the burden of phytopharmaceuticals (Li et al.).

In conclusion, it is currently rather difficult to make generalisations because available data are fragmentary and insufficient. More comprehensive and larger-scale studies are necessary to evaluate the occurrence and diversity of antimicrobials and AMR in wildlife and the environment. Within the One Health framework, it is crucial to understand the role played by wildlife and environment in the development and transmission of antimicrobial resistance, and to evaluate its impact.

## Author Contributions

All authors listed have made a substantial, direct and intellectual contribution to the work, and approved it for publication.

## Conflict of Interest

The authors declare that the research was conducted in the absence of any commercial or financial relationships that could be construed as a potential conflict of interest.

## Publisher's Note

All claims expressed in this article are solely those of the authors and do not necessarily represent those of their affiliated organizations, or those of the publisher, the editors and the reviewers. Any product that may be evaluated in this article, or claim that may be made by its manufacturer, is not guaranteed or endorsed by the publisher.

